# Valproate monotherapy induced-delirium due to hyperammonemia: A report of three adult cases with different types of presentation

**DOI:** 10.4103/0019-5545.42400

**Published:** 2008

**Authors:** R. Johnson Pradeep

**Affiliations:** Senior Resident, Department of Psychiatry, St. John's Medical College and Hospital, Bangalore, India

**Keywords:** Hyperammonemia, plasma ammonia, valproate-induced delirium

## Abstract

One of the important adverse effects of valproate is delirium due to hyperammonemia. In this case report series, we are reporting three cases with interesting and different types of clinical presentation on valproate monotherapy. Valproate-induced delirium may be mistaken for psychosis or worsening of mania leading to improper diagnosis and poor management. We found that there was an elevated level of plasma ammonia in our cases during the delirious state and which decreased when valproate was discontinued. In our cases the serum valproate levels, liver function tests, electroencephalogram, and imaging studies were normal, which were deranged in the previous case reports. We are the first to rechallenge valproate in one of the cases, to prove that valproate caused the hyperammonemia. We suggest that plasma ammonia levels should be monitored routinely in all cases of altered mental status and receiving valproate therapy.

## INTRODUCTION

Valproate (2-propyl pentanoic acid) is used commonly as an anti-convulsant and as a mood stabilizer in mania and bipolar disorders. Valproate-induced delirium due to hyperammonemia (VIDH) is one of the rare and interesting adverse effects seen in patients on valproate therapy. Even though previous case reports have reported VIDH, usually the patients were on additional medicines or there would be changes in the liver function tests (LFT), electroencephalogram (EEG), or imaging studies. Only eight genuine cases of VIDH have been reported previously. Here we are reporting three cases with different types of presentation on valproate monotherapy and without any changes in LFT, EEG, or imaging studies; which may cause diagnostic dilemma for busy clinicians. Also we are the first to have rechallenged valproate in one of the cases to prove that valproate has caused the delirium.

## CASE REPORT

### Case 1

A 53-year-old male with bipolar affective disorder on sodium valproate 1000 mg increased 2 days back presented to the emergency department. He was found to be talking excessively and irrelevantly, difficulty in walking, altered sleep pattern, and disorientation. He was unable to recognize his relatives, suspicious, and drowsy at times. Investigations were done to rule out any organic causes. Blood sugars, serum electrolytes, renal functions, LFTs, and sodium valproate levels (*n* = 50-100 µg/ml) were done and found to be normal. He was observed for 3 days without any medications and supportive care. On the third day of hospitalization patient improved and was found to be oriented, walking without any gait disturbances, and talking relevantly. A neurology consultation was taken and EEG was suggested to rule out a seizure disorder. However, the EEG was normal and the patient was restarted on valproate since there were no signs of toxicity. Initially, 400 mg on divided dosage was started and gradually increased to 1000 mg on subsequent days. On increasing, the dose to 1000 mg, he became ataxic again; was talking irrelevantly, had disturbed sleep and was found to be disoriented. Immediately, valproate was withheld and investigations such as CT scan, LFT, serum electrolytes, valproic acid levels, and plasma ammonia were done. All the investigations were found to be normal except a raised level of plasma ammonia to 95 µmole/l (*n* = 11-32 µmole/l). Patient improved after 3 days and plasma ammonia was repeated and was found to have decreased to 25 µmole/l.

### Case 2

A 60-year-old male with bipolar affective disorder was on valproate 1000 mg for a period of 1 month. He presented to the emergency department with complaints of vomiting, generalized weakness, and altered mental status for 3 days. The vomiting was nonbilious, nonprojectile, without blood and contained only food particles. There was no history of fever, headache, loss of consciousness, seizures, weakness of limbs, diabetes mellitus, or hypertension prior to the altered mental status. He was disoriented, without any focal deficits, hemodynamically stable, and other systems were normal. An initial diagnosis of delirium was made. Initial investigations such as hemogram, blood urea, creatinine, serum electrolytes, serum valproic acid, LFTs, EEG, and computer tomography of brain were found to be normal but serum ammonia was found to be elevated to 150 µmole/l (*n* = 11-32 µmole/l). Valproate was stopped. Patient stopped vomiting on the third day. He was oriented, talking relevantly, and generalized weakness also improved. Serum ammonia was repeated on the third day and it was decreased to 80 µmole/l (*n* = 11-32 µmole/l).

### Case 3

A 20-year-old male with seizure disorder was on Valproate 750 mg for a period of 3 years. He presented to the neurology OPD with drowsiness, altered sensorium, talking irrelevantly, and unsteadiness of gait for a period of 15 days. He also reported that he could see people, who were pricking him with a stick and was hearing people talking bad about him. In the ward also, he was found to be suspicious of people and at times aggressive. He was found to have disorientation to time, place and person, without any focal deficits, hemodynamically stable, and other systems were normal. Investigations such as cerebrospinal fluid studies, CT and MRI of the brain, EEG, hemogram, LFT, calcium, magnesium, and valproic acid were found to be normal and serum ammonia was elevated to 366 µmole/l (*n* = 11-32 µmole/l). Following the cessation of valproate and introduction of another antiepileptic, patient improved significantly in terms of altered mental status, aggression, auditory hallucinations, and gait disturbances by the fourth day.

## DISCUSSION

Valproate-induced hyperammonemia are usually a transient and asymptomatic phenomenon but can become chronic if undetected. It could manifest as lethargy, disorientation, and reversible cognitive deficits, which may progress to marked sedation, coma, and even death. VIDH without hepatic dysfunction is an adverse effect that has been reported extensively in pediatric population.[1] Even though many cases of VIDH have been reported, usually these patients have been on additional medicines such as topiramate or phenytoin added to valproate.[2,3] Only few adult cases of VIDH have been reported at therapeutic valproate levels.

Some of the common features in the above case reports are that all the three patients were receiving valproate monotherapy. The symptoms gradually decreased as soon as the valproate was discontinued. The plasma ammonia significantly corresponded to the symptoms and normalized as the symptoms resolved even though the type of presentations were different. All the relevant lab investigations, imaging studies, and electrophysiological studies were normal during the symptoms and during the resolution. We were the first to rechallenge valproate in one of the patients, as seen in Case 1 to prove that valproate caused the delirium due to hyperammonemia in spite of other laboratory investigations and imaging studies being normal. However, previous case reports have shown a consistent or a mild elevation in LFT and “triphasic waves" in the EEG.[4] Previous case reports have also shown this adverse effect to occur mainly during the initiation of the therapy but in our patient it occurred even on stable dosing, as seen in Case 2. In our patients, the most probable cause of delirium was valproate-induced hyperammonemia.

Different authors have proposed various mechanisms of action for VIDH. First mechanism of hyperammonemia is valproate-induced carnitine deficiency (by decreasing its biosynthesis) and inhibition of carbamoyl phosphate synthetase enzyme, which is the first enzyme of the urea cycle and suggested that replacement of carnitine as a treatment option.[5,6] Another mechanism proposed is by inhibition of glutamate uptake by astrocytes, leading to neuronal injury and perhaps cerebral edema. Glutamine production is increased while its release is inhibited causing increased intracellular osmolality, promoting an influx of water with resultant astrocyte swelling. This swelling could compromise astrocyte energy metabolism and results in cerebral edema, and increased intracranial pressure.[7] Kidneys also contribute to the VIDH by increasing glutamate uptake and ammonia release into serum from the kidneys by glutaminase stimulation in the renal mitochondria by valproate.[8] Concomitant use of other antiepileptics like topiramate, aspirin, and cimetidine may elevate the free fraction of valproate leading to hyperammonemia.[9] Comorbid renal failure and hypoalbuminemia also contribute to VIDH.

In conclusion, clinicians should consider hyperammonemia in all patients with delirium or mental status changes receiving valproate monotherapy treatment. Busy clinicians can mistake it for worsening of manic symptoms or increased sedation. Plasma ammonia levels should be done in addition to renal and LFTs in these patients. Discontinuation of valproate should be considered for prompt reversal of symptoms. Dietary carnitine supplementation (2-4 g/day) may be helpful both in the prevention and in the treatment of hyperammonemia secondary to valproate but further studies are required to confirm the same.[10]
